# Functional-oriented, portable brain–computer interface training for hand motor recovery after stroke: a randomized controlled study

**DOI:** 10.3389/fnins.2023.1146146

**Published:** 2023-05-11

**Authors:** Jianghong Fu, Shugeng Chen, Xiaokang Shu, Yifang Lin, Zewu Jiang, Dongshuai Wei, Jiajia Gao, Jie Jia

**Affiliations:** ^1^Department of Rehabilitation Medicine, Huashan Hospital, Fudan University, Shanghai, China; ^2^School of Mechanical Engineering, Shanghai Jiao Tong University, Shanghai, China; ^3^Department of Rehabilitation Medicine, Shanghai No. 3 Rehabilitation Hospital, Shanghai, China; ^4^National Clinical Research Center for Aging and Medicine, Huashan Hospital, Fudan University, Shanghai, China; ^5^National Center for Neurological Disorders, Huashan Hospital, Fudan University, Shanghai, China

**Keywords:** brain-computer interfaces, hand rehabilitation, stroke, motor recovery, RCT

## Abstract

**Background:**

Brain–computer interfaces (BCIs) have been proven to be effective for hand motor recovery after stroke. Facing kinds of dysfunction of the paretic hand, the motor task of BCIs for hand rehabilitation is relatively single, and the operation of many BCI devices is complex for clinical use. Therefore, we proposed a functional-oriented, portable BCI equipment and explored the efficiency of hand motor recovery after a stroke.

**Materials and methods:**

Stroke patients were randomly assigned to the BCI group and the control group. The BCI group received BCI-based grasp/open motor training, while the control group received task-oriented guidance training. Both groups received 20 sessions of motor training in 4 weeks, and each session lasted for 30 min. The Fugl–Meyer assessment of the upper limb (FMA-UE) was applied for the assessment of rehabilitation outcomes, and the EEG signals were obtained for processing.

**Results:**

The progress of FMA-UE between the BCI group [10.50 (5.75, 16.50)] and the control group [5.00 (4.00, 8.00)] was significantly different (*Z* = −2.834, *P* = 0.005). Meanwhile, the FMA-UE of both groups improved significantly (*P* < 0.001). A total of 24 patients in the BCI group achieved the minimal clinically important difference (MCID) of FMA-UE with an effective rate of 80%, and 16 in the control group achieved the MCID, with an effective rate of 51.6%. The lateral index of the open task in the BCI group was significantly decreased (*Z* = −2.704, *P* = 0.007). The average BCI accuracy for 24 stroke patients in 20 sessions was 70.7%, which was improved by 5.0% in the final session compared with the first session.

**Conclusion:**

Targeted hand movement and two motor task modes, namely grasp and open, to be applied in a BCI design may be suitable in stroke patients with hand dysfunction. The functional-oriented, portable BCI training can promote hand recovery after a stroke, and it is expected to be widely used in clinical practice. The lateral index change of inter-hemispheric balance may be the mechanism of motor recovery.

**Trial registration number:**

ChiCTR2100044492.

## Background

Stroke causes the highest morbidity associated with disability-adjusted life years lost in China, with 2 million new cases annually (Wu et al., 2019). Approximately 66% of stroke survivors experience upper limb motor impairments, which results in functional limitations in activities of daily living and leads to a low life quality (Kwah et al., 2013; Morris et al., 2013) and increases the burden for family and society. Hand function rehabilitation is a research hotspot and a challenge in the field of stroke rehabilitation. Among all the neural modulation technologies, brain–computer interfaces (BCIs) have been proven to be effective for hand motor recovery after stroke (Biasiucci et al., 2018; Cervera et al., 2018; Baniqued et al., 2021).

The workflow of BCI includes acquiring brain signals, extracting features, transforming the signal into command via external devices, and activating the sensory feedback. The BCI equipment has been updated from a fixed-position device to a mobile one (Mattia et al., 2020). However, a portable BCI device may be more flexible for use in stroke rehabilitation. In a typical electroencephalography (EEG)-based non-invasive BCI, the user's movement intention such as motor imagery (MI) or motor attempt (MA) is decoded in real time from the ongoing electrical activity of the brain by extracting relevant features (Cervera et al., 2018). Many BCI-based motor rehabilitation systems traditionally encompass neural activity decoders of ipsilesional sensorimotor activity (sensorimotor rhythm, SMR, 9–15 Hz) (Cervera et al., 2018). SMRs can be measured over the sensorimotor cortex (SMC) and modulated by MI, MA, or motor execution (ME) tasks (Frenkel-Toledo et al., 2014; Yuan and He, 2014). Task-related modulation in EEG-based SMRs is usually manifested as ERD or ERS in low-frequency components [mu rhythm (8–12 Hz) and beta rhythm (13–26 Hz)] (Pfurtscheller and Lopes, 1999). MI or MA is associated with ERD of mu rhythm oscillations recordable over SMC (electrode sites C3 and C4) using EEG (Hasegawa et al., 2017; Remsik et al., 2019). MI is a mental activity in which a specific movement is performed in the mind without actual movement (Kilteni et al., 2018). MA is an attempt of the paralyzed limb to move while there is still no actual or little movement, but the electromyography activity in the affected arm is several orders higher in the motion phase than in the rest phase (Antelis et al., 2017). They were both used extensively in the BCI experiment as an active way of neuromodulation. ME was mostly used in healthy participants (Meng et al., 2018; Chen et al., 2019). Specifically, a meta-analysis demonstrated that motor attempt-based BCIs seem to be more effective than MI-based BCIs (Bai et al., 2020). MA-based BCIs had superior effects compared to MI-based BCIs. The SMR-based BCIs detect characteristic changes in SMC in response to the motor task, and the paradigm was adopted in several studies (Robinson et al., 2018; Li et al., 2021; Pinter et al., 2021). In a study by Biasiucci et al. (2018), they asked the patients to attempt to extend the affected hand (fingers and wrist) as the motor task; Chen et al. (2020) designed an extension of the wrist as the motor task; and Ramos-Murguialday et al. (2013) instructed their patients to try to reach (even if the arm does not follow their intention), grasp, and bring an imaginary apple to their lap, and finger extension was involved in the reaching and grasping movement. They all obtained functional improvement.

Currently, in clinical trials of BCI for basic motor training, only a single motor task is designed, which is not able to fully meet clinical requirements. Many stroke patients in the acute stage undergo flaccid paralysis stage, and they can hardly move their hands, let alone do a grasp or open movement. Many patients may go through the synergetic motion mode stage, where they can perform different levels of grasping activity. Similarly, a segregation movement like the hand open motion is also a hard step; full extension of affected fingers would mean great progress in recovery. In total, many of them conform to the recovery of the Brunnstrom I–VI recovery stages (Naghdi et al., 2010). Since grasp and open movements are fundamental but essential hand motions, imaging the grasp (Ang et al., 2014) or/and extension (Pichiorri et al., 2015) of the affected hand was designed in many BCI studies. The common motor tasks adopted in the BCIs experiment included the movements of the proximal joints, shoulder, elbow, wrist, and hand, especially for the distal joints. Simultaneously, the feedback provided by robotic devices used in upper limb rehabilitation exists in the form of exoskeletons or end effectors. Robotic exoskeletons (i.e., powered orthoses or braces) are wearable devices where the actuators are biomechanically aligned with the wearer's joints and linkages, allowing the additional torque to provide assistance, augmentation, and even resistance during training (Molteni et al., 2018; Baniqued et al., 2021). In comparison, end-effector systems generate movement by applying forces to the most distal segment of the extremity via handles and attachments (Molteni et al., 2018; Baniqued et al., 2021). The recovery of the paretic hand is a harder step and a core component in regaining functional movement. The hand tasks consisted of both simple and complex forms. Some complex forms include not only basic movements but also movements related to daily life. Despite patients' varied functional needs, many brain–computer interface studies set only one motor task. In a study by Ramos-Murguialday et al. (2019), they applied two kinds of motor tasks with the assistance of robots, for patients, namely the upper limb and the paretic hand, and the number of sessions with hand or arm movements was balanced between groups. Still, few of them have applied the different motor tasks to BCIs experiments as patients needed.

Plow et al. (2015) pointed out that corticospinal plasticity, return of balance between excitability of the ipsilesional and contralesional motor regions, and vicarious recruitment of widespread frontal and parietal synergistic regions promote the clinical recovery of paretic hand function, and other researchers assumed that the regrowth of ipsilateral descending fibers from the unaffected hemisphere to denervated motor neurons may contribute to the recovery (Gao et al., 2022). They both reflected that the balance of the two hemispheres may be the key to recovery after a stroke. The laterality index (LI) was considered the normalized difference between brain activations in the left and the right hemispheres (Caria et al., 2011). Therefore, facing the current situation of the single motor task in BCI study and the difficulty in clinical promotion, we propose two kinds of motor tasks for hands by the robot for different patients and to testify the functional-oriented, portable BCI training with an EEG-based hand robot for hand recovery after stroke and explored the balance changes in brain activations of the bilateral hemispheres.

## Methods

### Participant recruitment and randomization

**Figure 1:**
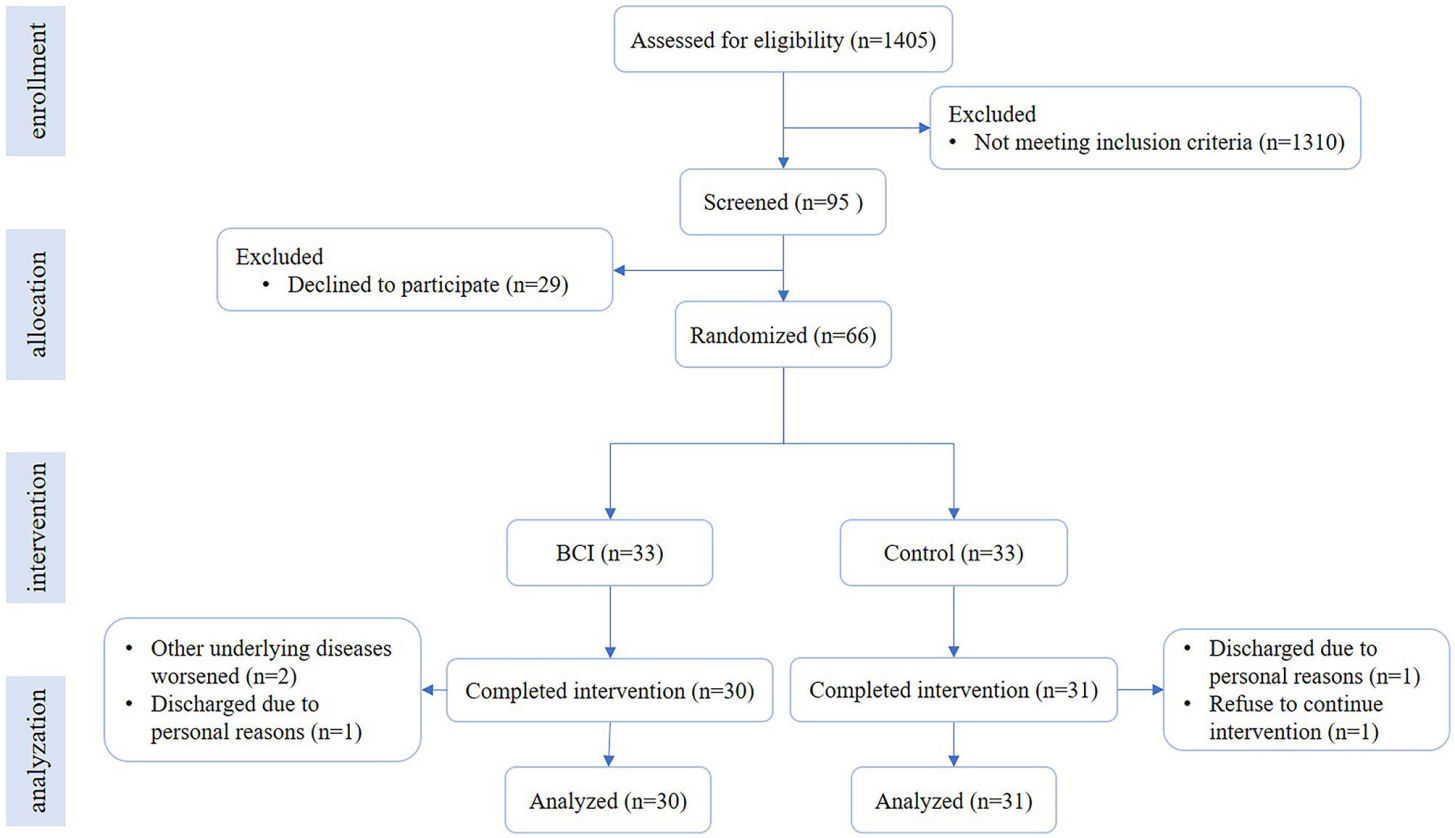
Flowchart of the trial. The flowchart shows the process of recruitment, grouping, intervention, and data analysis.

We conducted a randomized controlled trial. The randomization allocation sequence was 1:1, the allocation information (BCI group and control group) was sealed in opaque envelopes, and each enrolled patient was randomly selected. Enrollment and assignment of participants were performed by one researcher. As participant blinding was not feasible, all outcome assessments were performed by an experienced therapist, who was blinded to allocation. Figure 1 shows the flowchart of the trial. Stroke patients were recruited from the Department of Rehabilitation Medicine, Huashan Hospital, and the Department of Rehabilitation Medicine, Jing'an District Central Hospital of Shanghai, from February 2021 to December 2022. The inclusion criteria for patients following stroke were as follows: (1) ischemic or hemorrhagic stroke diagnosed through computed tomography or magnetic resonance imaging (MRI); (2) age in the range of 18–80 years; (3) at least 2 weeks since stroke onset and less than 1 year; (4) Brunnstrom stages of paretic hand are I–V; (5) mini-mental state examination ≥20 scores, able to obey basic commands; and (6) able to sit on a chair independently for at least 1 h. The exclusion criteria were as follows: (1) having a cardiac pacemaker; (2) pregnancy; (3) allergy to EEG electrode cream; (4) any osteoarthrosis (including joint deformity) that could cause joint contracture in the hand or upper limb; and (5) unstable fracture in the paretic upper limb. Written informed consent was provided by all participants. This study was approved by the Ethical Committee of Huashan Hospital [(2021) Provisional Examination No. (039)] and was performed according to the Declaration of Helsinki, and the trial was registered at the Chinese Clinical Trial Registry (ChiCTR2100044492).

### Primary and secondary outcomes

The primary clinical outcome of the study was the change in the Fugl–Meyer assessment of the upper limb (FMA-UE), with a total score of 0 to 66, which was used to assess the severity of motor dysfunction. Secondary outcomes included EEG data and BCI accuracy. Clinical evaluations were performed immediately before and after the intervention.

### Characteristics of the enrolled patients

A total of 66 patients were enrolled in this study. They were randomly allocated into the BCI group (*n* = 33) and control group (*n* = 33), and 30 cases in the BCI group and 31 cases in the control group completed the intervention. The average age was 55.93 ± 11.05 years old in the BCI group and 59.00 ± 14.49 years old in the control group. There were 23 (76.7%) male patients in the BCI group and 24 (80.6%) male patients in the control group. The infarction cases were 24 (80.0%) in the BCI group and 24 (77.4%) in the control group. The average course was 77.50 (33.75, 175.25) d in the BCI group and 64.00 (37.00, 150.00) d in the control group. The details are in Table 1, and there was no significant difference in the demographic characteristics between the two groups. Additional details are provided in Supplementary Table 1.

**Table 1:** Characteristics of the enrolled patients.

**Item**	**BCI group (*n* = 30)**	**Control group (*n* = 31)**	***t*/χ^2^/*Z***	***P*-values**
Age (MD ± SD)	55.93 ± 11.05	59.00 ± 14.49	−0.927	0.357
Male/(%)	23 (76.7%)	25 (80.6%)	0.144	0.704
**Type**				
Infarction	24 (80.0%)	24 (77.4%)	0.061	0.806
Hemorrhage	6 (20.0%)	7 (22.6%)		
Course/d	77.50 (33.75, 175.25)	64.00 (37.00, 150.00)	−0.390	0.697

### EEG recording

Participants were asked to sit in a chair in front of a computer screen. An EEG cap was used to record EEG signals. A total of 10 channels of Ag/AgCl electrodes were distributed according to the 10–20 system. The reference channel and the ground channel were placed, respectively, on the right mastoid process and the forehead. The impedance of electrodes was kept <5 kΩ. EEG signals were amplified with the CommercialAmp (iRecorder W16, Niantong Intelligence Ltd., China) and recorded at a sampling rate of 500 Hz.

### Feature extraction and classification

**Figure 2:**
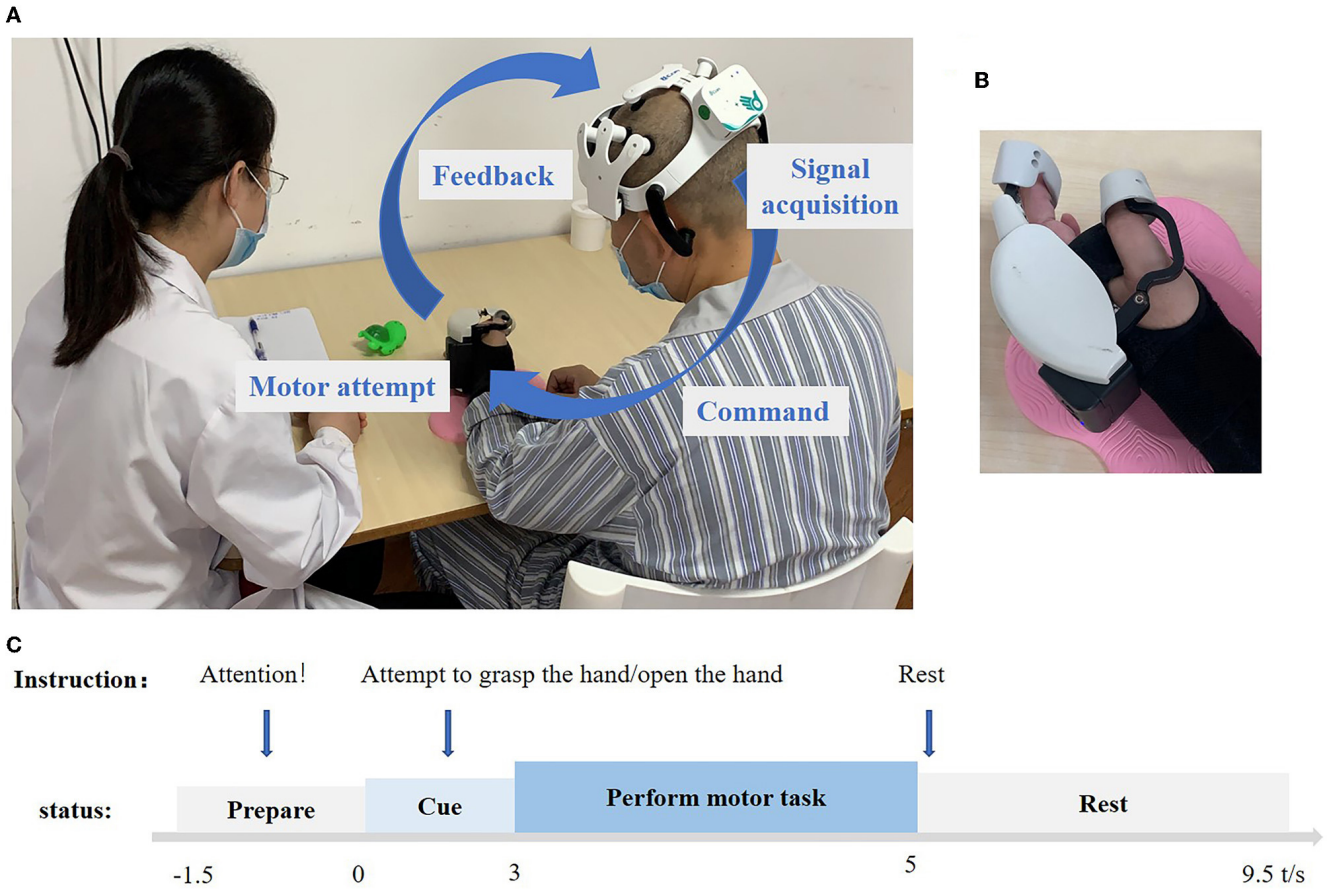
**(A)** Overview of the BCI system pipeline. The device is mainly composed of an amplifier, EEG cap, and hand robot. A 10-channel electroencephalogram (EEG) device was used to record the movement intention of the subjects in real time, and the movement commands were analyzed by artificial intelligence algorithms and sent to the hand robot wirelessly. The signal parsing part adopts the on-chip decoding technology, which can be run independently from the computer. The hand robot part uses a combination of the rigid transmission mechanism and flexible fixation material, which can be worn by the patient with one hand to assist the complete grasping and stretching movements. The maximum output of grasping force is 5 N, and it can be connected with the EEG cap part with a one-button connection. **(B)** Hand robot. **(C)** Experimental setup of one trial. −1.5 to 0 s voice commands for preparation, subjects' state for resting preparation, 0 to 3 s voice commands for attempting to open the hand or attempting to grasp the hand, patients begin to prepare to do the action, 3–5 s, no voice commands, patients perform the motor task, 5– 9.5 s, voice commands for rest, and subjects also rest.

In this study, a common spatial pattern (CSP) (Ramoser et al., 2000) was used for feature extraction, and the log variance of the first and last two components produced by CSP filters were selected as feature vectors (Shu et al., 2018). The method of linear discriminative analysis was employed for discriminating different tasks (MA vs. Rest). The pattern classifications were conducted online with 10 channels of EEG signals. EEG features were extracted from the time segment of [3 5] s and frequency band of [8 30] Hz. This time segment can be viewed in Figure 2C (performance of the motor task).

### BCI group

#### EEG calibration

Before training, the patients would go through EEG calibration for each task, and the voice instructions were “attempt to grasp, rest,” and “relax, rest” 10 times but in a random order. After 20 trials for calibration (Shu et al., 2018), BCI training was then performed.

#### BCI training

Patients were instructed to undergo two kinds of MA training: hand grasping and opening. During the grasp training process, the instruction for training was “attempt to grasp, rest” for each trial, which lasted for 11 s and repeated 30 times for each session and three sessions of one-time training. As for the open task, the training was the same process, the only difference being the instruction, “attempt to open, rest.” This was recycled 30 times for each session, with three sessions for one-time training. During the training, we paid close attention to their behavioral movements and corrected the movement of other body parts in a timely manner. The training was arranged one time a day, 30 min foat a time, 5 days for a week, for 4 weeks, and 20 times in total. The grasp/open task was trained once each time. The BCI system setup and trial pipeline can be seen in Figure 2.

### Feedback-hand robot

Wearable hand exoskeletons were used for motor feedback. The patients wore a robot, and the EEG rhythms in the sensorimotor area of the brain were detected to control the opening and grasping of a robotic hand. When the intention of the attempt to grasp/open was successfully detected, the robot could assist the movement of the paretic hand.

### Control group

They received motor task-based task-oriented training guidance, including grabbing a block of wood, toy grasp, and release. Even some severely injured patients can wear robots to assist their hand movement. The training was arranged once a day, 30 min a session, 5 days for a week, for 4 weeks, with 20 sessions in total. The two group patients also received 20 min of physical therapy, 20 min of occupational therapy, and 20 min of neuromuscular electrical stimulation.

### EEG processing

The EEGLAB v2021.1 and MATLAB R2021a were used in the EEG analysis. EEG data from 10 channels were used in processing. The left hemisphere was covered with FC3, CP3, C1, C3, and C5 (five channels) while the right with FC4, CP4, C2, C4, and C6 (five channels). The preprocessed EEG data consisted of high-pass filtering at 8 Hz and low-pass filtering at 30 Hz. Stop-band filter filtering was 49–51 Hz. Since the artifacts might still exist between 8 and 30 Hz, the frequency of artifactual oscillations often overlaps with the interest frequency; thus, the independent component analysis components representing eyeblink, head movement, and power line interference were removed from the data (Goncharova et al., 2003; Fatourechi et al., 2007). Manual checking was performed in the EEG data of all 10 channels and all trials. The power spectrum of all 10 channels was computed at the frequency of alpha (8–13 Hz) to identify ERD on grasp and open tasks. Time–frequency distributions of EEG trials were estimated using a windowed Fourier transform (WFT) with a fixed 400 ms Hanning window. WFT yielded, for each trial, a complex time–frequency estimate *F*(*t, f*) at each time–frequency point (*t, f*), extending from −1,500 to 8,500 ms (in steps of 2 ms) in the time domain and from 8 to 30 Hz (in steps of 1 Hz) in the frequency domain. Power spectrum (*P*), *P*(*t, f*) = |*F*(*t, f*)|2, was obtained. The percentage of relative power change was calculated to obtain the ERD concerning a resting-state baseline ([−1.5, 0] s). The interest time was set both at [3, 5] s after the cue [0, 2] s of the event. During the [3, 5] s, the patient was performing the MA tasks. The power spectrum of interest in the period after the event is given by A whereas that of the preceding baseline period is given by *R*. ERD or ERS was calculated according to the equation:


$$\begin{array}{l} \text{ERD}/\text{ERS }=\;\left(\text{A }-\text{ R}\right)/\text{R}\times 100\text{\%}. \end{array}$$

Under this definition, ERD was usually expressed as a negative value, while ERS was usually expressed as a positive value. The time–frequency maps were drawn with the above mentioned calculation, representing the signal magnitude as a joint function of time and frequency at each time–frequency point. The topographies were drawn with an interesting time of 3–5 s, concerning a resting-state baseline ([−1.5, 0] s). We calculated the ERD from the affected hemisphere (C3/C4) of the brain and obtained the average power spectrum for all 30 trials. The laterality index (LI), expressed as the normalized difference between brain activation in the left and the right hemisphere, approached a value of 1 or −1 when the activity was either purely contralesional or ipsilesional (Caria et al., 2011; Chen et al., 2021), and we calculate by the ERD from C3/C4 electrode according to the equation:


$$\begin{array}{l} \text{LI }=\left(\text{ER}{\text{D}}_{\text{ipsilesiional}}-\text{ER}{\text{D}}_{\text{contralesional}}\right)/\\ \;\left(\left|{\text{ERD}}_{\text{ipsilesiional}}|+|ERD_{contralesional}\right|\right).\; \end{array}$$

### Statistical analysis

#### Sample size

The sample size was calculated based on the primary outcome, that is, BCI intervention is superior in improving the FMA-UE. Based on preliminary findings effect size *d* = 0.8, the alpha level at 5%, statistical power at 80%, the ratio between the two groups was 1:1, G power 3.1 was used to calculate the sample size, and 26 patients per group was needed, assuming a drop-out rate 20%, and 33 participants per group were enrolled in the study.

The statistical analysis was performed with SPSS version 26.0 (SPSS Inc.) and figures were drawn with GraphPad Prism 8 (GraphPad Software, Inc.). Kolmogorov–Smirnov tests were first applied to check the normality of the variables. For Gaussian variables, data were expressed as mean ± standard deviation, and for non-Gaussian variables, they were expressed as median (P25, P75). The chi-square test was used for binary variables, such as gender and diagnosis (Infarction/hemorrhage); the *t*-test was used for Gaussian variables, such as the age of patients; otherwise, a non-parametric test (the course of the disease) was used for basic feature descriptions. Since FMA-UE and LI do not follow a normal distribution, paired non-parametric rank-sum tests (Wilcoxon rank-sum test) were used before and after the intervention for each group, and independent-sample non-parametric rank-sum tests (Wilcoxon rank-sum test) were used for the two groups' analysis. Studies have shown that 4.6 scores reached the minimal clinically important difference (MCID) (Chen Ruiquan, 2015); thus, we calculated the effective rate by dividing the number of MCID reached by each group by the total number. Statistical significance was set at a *P*-value of < 0.05 (two-tailed).

## Results

### Rehabilitation outcome of the patients

**Figure 3:**
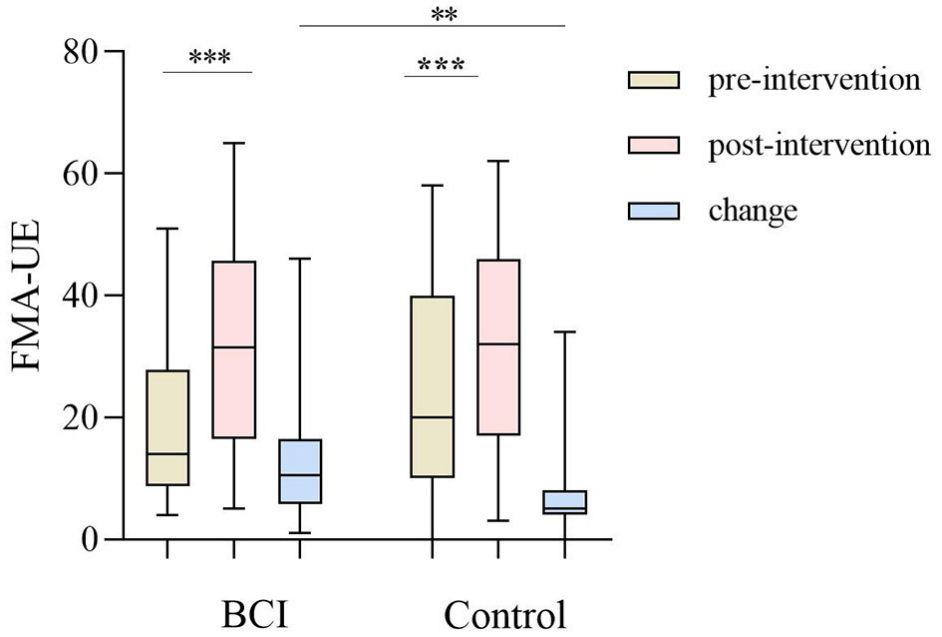
The upper limb of the Fugl–Meyer assessment (FMA-UE) improved between the BCI group (*P* < 0.001) and the control group (*P* < 0.001) before and after the intervention, and the progress between the BCI group and control group of FMA-UE (*P* = 0.005). The line shows the median (P25, P75). ^**^P < 0.01; ^***^P < 0.001.

The change of FMA-UE between the BCI group [10.05 (5.75, 16.50)] and the control group [5.00 (4.00, 8.00)] was significant (*Z* = −2.834, *P* = 0.005; Table 2). After 1 month of training, the FMA-UE of both groups improved significantly (*P* < 0.001). Before the intervention, the FMA-UE in the BCI group was 14.00 (8.75, 27.75), compared with the control group's 20.00 (10.00, 40.00), and there was no significant difference between the two groups (*Z* = −1.364, *P* = 0.173) at the baseline; while after the intervention the FMA-UE in the BCI group was 31.50 (16.50, 45.75), compared with the control group 32.00 (17.00, 46.00), and there still was no significant difference between the two groups (*Z* = −0.115, *P* = 0.908; Figure 3, Table 3). A total of 24 patients in the BCI group achieved the MCID of FMA-UE with an effective rate of 80%, and 16 in the control group achieved the MCID, with an effective rate of 51.6%.

**Table 2:** Progress of the FMA-UE for the two groups.

**Item**	**BCI group (*n* = 30)**	**Control group (*n* = 31)**	***Z***	***P*-values**
ΔFMA-UE	10.50 (5.75, 16.50)	5.00 (4.00, 8.00)	−2.834	0.005

ΔFMA-UE represents the changes of FMA-UE before and after the intervention, and it does not follow a normal distribution; these data are expressed as the median (P25, P75).

**Table 3:** FMA-UE for the two groups before and after the intervention.

**Item**	**BCI group (*n* = 30)**	**Control group (*n* = 31)**	***Z***	***P*-values**
Pre	14.00 (8.75, 27.75)	20.00 (10.00, 40.00)	−1.364	0.173
Post	31.50 (16.50, 45.75)	32.00 (17.00, 46.00)	−0.115	0.908
*Z*	−4.784	−4.772		
*P*-values	<0.001	<0.001		

Since FMA-UE does not follow a normal distribution, these data are expressed as the median (P25, P75).

### The lateral index changes

**Figure 4:**
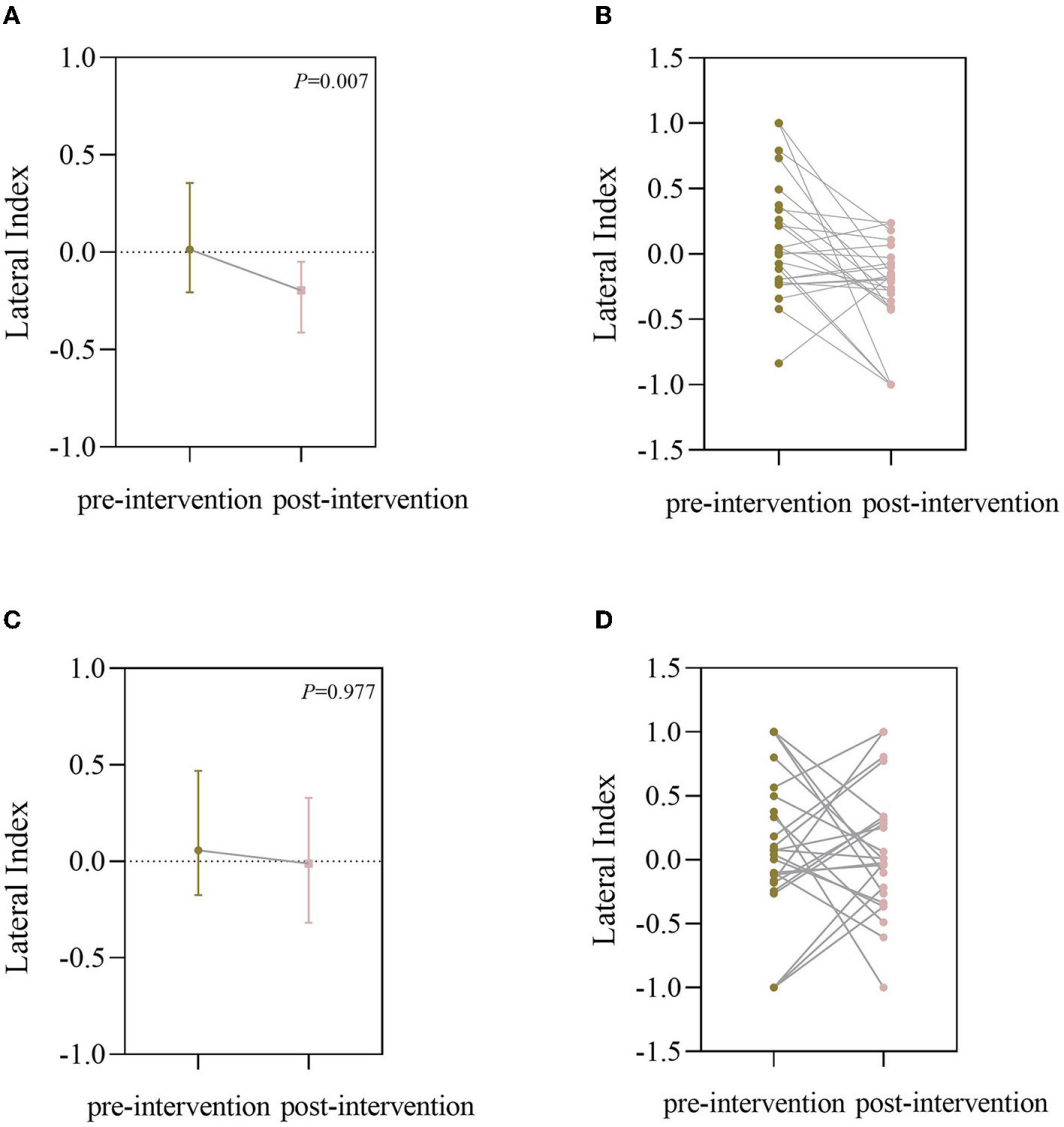
**(A)** Lateral index (LI) of the BCI group for the open task (it was plotted as a median with an interquartile range); **(B)** LI of individual changes for the open task; **(C)** LI of the BCI group for the grasping task (it was plotted as median with interquartile range); **(D)** LI of individuals changes for the grasping task.

**Figure 5:**
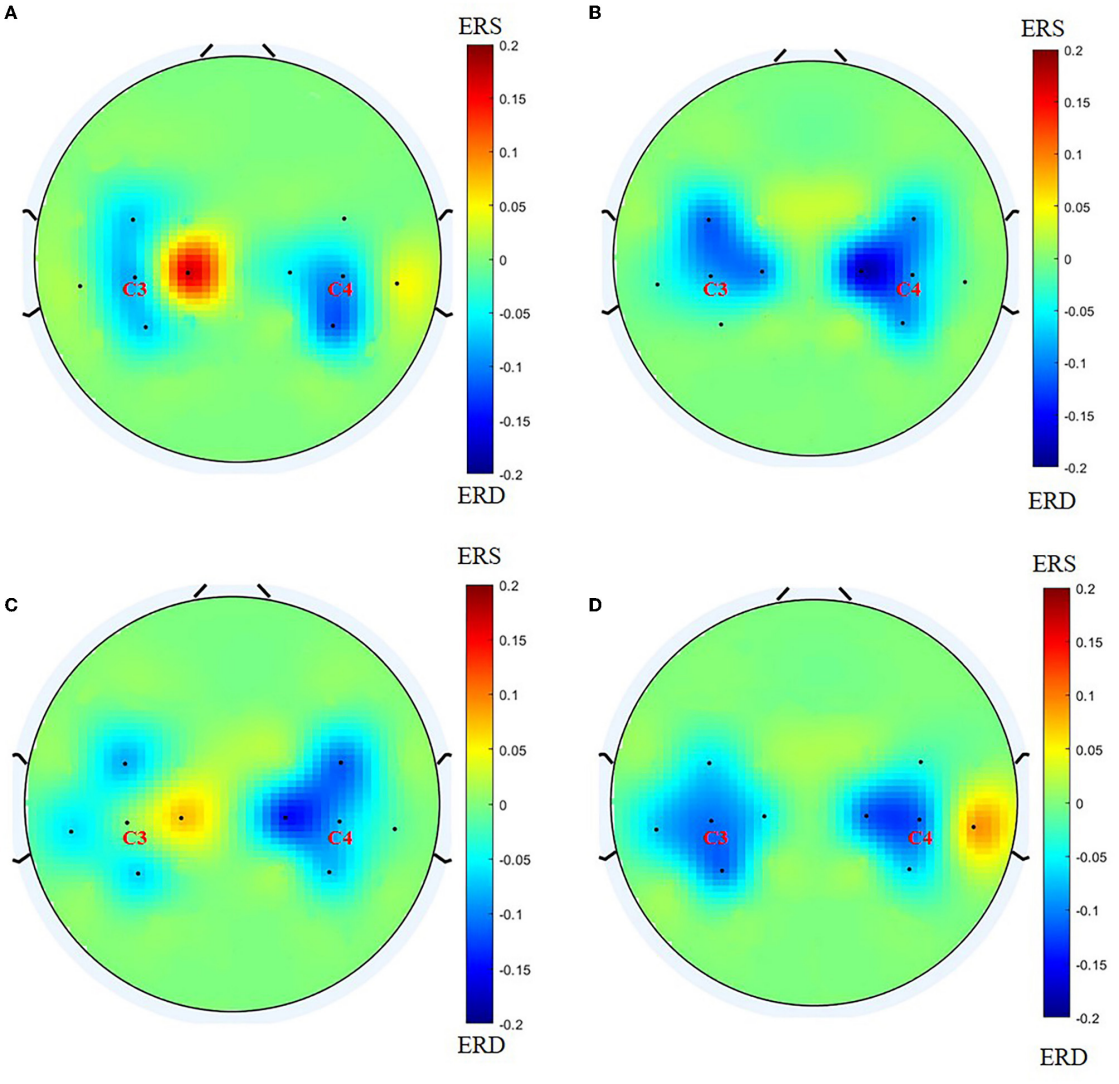
Before drawing the topology, we flip the lesion so that the affected side is on the left side. **(A)** Topography for grasping task before training in the BCI group; **(B)** topography for grasping task after training in the BCI group; **(C)** topography for the open task before training in the BCI group; **(D)** topography for the open task after training in the BCI group.

Before BCI training, the lateral index of the BCI group was 0.0567 (−0.740, 0.4688) for the grasping task and 0.0046 (−0.2108, 0.3650) for the open task, compared with post-BCI training, the lateral index of BCI group was −0.0109 (−0.3183, 0.3290) for the grasping task and −0.1909 (−0.3957, −0.0374) for the open task, and there was a significant difference for the open task (*Z* = −2.704, *P* = 0.007; Figure 4). Figure 5 shows the changes in the topological graph for the two motor tasks before and after the intervention. The lesioned side was flipped so that all the affected sides were on the left side. It shows the topographies for the grasping task before (Figure 5A) and after (Figure 5B) training in the BCI group and the topographies for the open task before (Figure 5C) and after (Figure 5D) training in the BCI group. After the BCI intervention, the lesioned hemisphere shows great activations (ERD) compared to the topography before the intervention. These results can be seen in both grasp and open tasks.

### BCI accuracy

**Figure 6:**
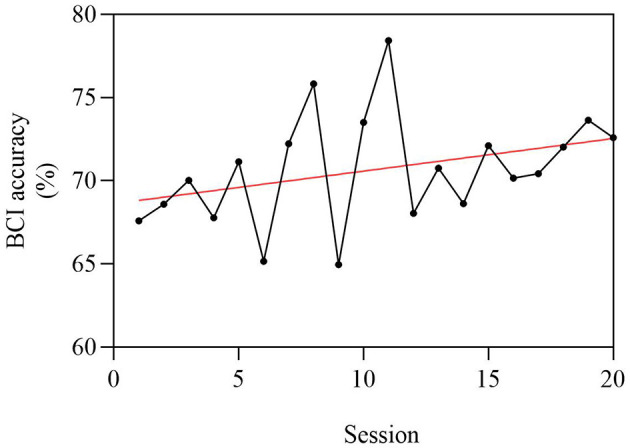
Average BCI accuracy of 24 stroke patients, with a total of 20 sessions. The abscissa is 1–20 times of BCI intervention, and the ordinate is the BCI accuracy. Since the average BCI accuracy of patients is between 60 and 80%, the ordinate is set to 60%−80%. The average BCI accuracy was 70.7% for 20 sessions, and it improved by 5.0% in the final session compared with the original session.

The average BCI accuracy for 24 strokes in BCI group patients was 70.7% for 20 sessions, and it improved by 5.0% in the final session compared with the original session (Figure 6). However, the improvement in BCI accuracy does not have a significant correlation with the improvement of the FMA-UE.

## Discussion

We explored the clinical efficacy of the functional-oriented MA-based BCI training for stroke patients after 1-month of training with hand robotic feedback, and they obtained more FMA-UE improvements than the control group after 20 sessions of BCI training. It indicated that functional-oriented BCI training can promote hand and upper limb motor recovery for stroke patients.

FMA-UE in the BCI group improved [10.05 (5.75, 16.50)] compared with the control group [5.00 (4.00, 8.00)]. They were all within 1 year of stroke onset, and they made up the majority of the inpatient ward population. Studies have shown that 4.6 scores reached the MCID (Chen Ruiquan, 2015), and our study shows that 80% can obtain functional improvement in the BCI group, and over half of the patients in the control group can obtain functional improvement. The importance of MCID has already been noticed in Ramos-Murguialday et al.'s (2013) study, and they considered a change in the range of 3.4 points on the modified FMA-UE (with a maximal score of 54 points) motor activity-related scores as a change from no activity to some muscles involved in lifting and stretching the arm, turning the forearm, and extending the wrist and/or fingers.

We carried out the functional-oriented training with our portable, wireless Bluetooth-connected BCI equipment for hand rehabilitation after stroke, and the results showed that the BCI group achieved significant progress. Several stroke patients in the acute stage could hardly move their hands, neither completely nor incompletely grasp. An improvement from no active movement to active movement is a hard step, and separation movement, being able to open the finger, is also a difficult process. Patients with moderate-to-severe brain injuries often have trouble in grasping or opening their hands. We assigned grasp and open tasks to different patients. If they have trouble with finger flexing or grasping the hand, they may need more grasp training; once they grasp the hand but have trouble with extending fingers, they may need more open training. Therefore, with different kinds of injured hands, we can induce the desired function to recover and reinforce the existing function by use of our BCI equipment.

Motor tasks are the main component in BCIs. In our study, we chose the MA as the mental task. We instructed patients to attempt to grasp or open the hand, and then, the hand robot can assist with the movement of the paretic hand as the feedback. We adopted motor attempts in our study; before training, we trained the patients on how to do the motor attempt movement, and in the training, our instruction was “attempt to grasp” or “attempt to open” with supervision, and they completed the motion smoothly. MA-based BCIs established a closed sensorimotor loop, which can potentially restore the normal timing order of motor preparation, execution, and peripheral muscle effectors, and this form of plasticity may further strengthen corticospinal tract projection (Bai et al., 2020).

The inter-hemispheric balance between the ipsilesional and contralesional motor cortices can return with recovery (Plow et al., 2015). As we all know, the bilateral hemispheres are in homeostasis through mutual transcallosal inhibition, but the stroke disrupted the balance. Contralesional areas instead intensify their inhibition upon the already weak ipsilesional, which explains poststroke dysfunction (Murase et al., 2004). In our study, we also investigated the LI by the EEG signal from electrodes C3/C4. LI can be considered as the normalized difference between brain activations in the left and the right hemisphere, approaching a value of 1 or −1 when the activity was either purely contralesional or ipsilesional. In Ramos-Murguialday et al.'s (2013) BCI study, they explored the LI of brain activity in the motor and premotor cortices during the “actual” movement condition under functional MRI and testified that FMA improvements in the experimental group correlated with changes in functional MRI laterality index. In our study, we instructed the patients to attempt to move the paralytic limb and calculated the LI from the C3/C4 by EEG, thus, the brain activations from both or contralesional to only the ipsilesional hemisphere may be a good sign for recovery. Therefore, the balance between bilateral hemispheres returned generally and may promote brain recovery and functional improvement. We found that the LI for the open task was improved after the BCI training, but there was no significant difference for the grasp EEG assessment even though there are some tendencies to decline. The average of the topological graph also demonstrated the changes before and after the intervention. It showed the change of brain activations from unaffected to both sides in the grasping task, and in the open task, the brain activations were from around the electrodes to the SMC areas also means a big step.

Many previous studies have explored the relationship between improvements in motor function and BCI accuracy. Some scholars considered that (Chen et al., 2020) stroke patients with good recovery showed relatively higher online BCI accuracy, and others showed a slight decline in hit rate over time even though their function was improved (Biasiucci et al., 2018). In our study, the average BCI accuracy was 70.7% for 24 patients (six patients were not calculated because the EEG data was ruined), and this is similar to existing studies (Biasiucci et al., 2018; Miao et al., 2020). We also found that the average BCI accuracy for patients was improved after BCI training, but there was no correlation with the function changes. Thus, a good BCI accuracy may be eligible for BCI training (Ahn et al., 2013), but it may not be an appropriate index to predict functional changes.

In brief, the use of 10-channel EEG could be a limitation in our study, and because of the limited channels, we cannot make further analysis, such as functional connectivity analysis. We did not obtain EEG from the control group, and it might influence the explanation of the LI for motor recovery. Although we have achieved positive results, in the future, we will carry out multi-center clinical trials on a larger scale across the country to recruit more stroke subjects to reach a more reliable conclusion. In addition, only two kinds of basic hand movement were adopted in our study and could be another limitation of our study since there are many types of dysfunction of the hand. In the future, we can design more motor tasks in the BCI experiment to benefit more patients.

## Conclusion

The functional-oriented BCI training can promote hand recovery after stroke, and the rebalance in brain activations of inter-hemispheres may be the mechanism of motor recovery. Due to its two motor task modes, portability, and Bluetooth-connected characteristics, it is expected to be widely used in the clinic.

## Data availability statement

The original contributions presented in the study are included in the article/Supplementary material, further inquiries can be directed to the corresponding author.

## Ethics statement

The studies involving human participants were reviewed and approved by Ethics Committee of Huashan Hospital. The patients/participants provided their written informed consent to participate in this study.

## Author contributions

JJ and JF designed the study. JF, YL, ZJ, DW, and JG performed the study. SC and XS organized the data and performed the data analysis. JF wrote the manuscript. SC, XS, and JJ reviewed and edited the manuscript. All authors read and approved the submitted manuscript.
