# Supplementary material for: Functional-oriented, portable brain–computer interface training for hand motor recovery after stroke: a randomized controlled study
Source: Front Neurosci. 2023 May 11;17:1146146. doi: 10.3389/fnins.2023.1146146 (PMC10213744; doi:10.3389/fnins.2023.1146146)
Supplement: Supplementary file 1 [file Table_1.DOCX]

Table1 Detailed characteristics of the subjects

| Subjects | gender | age | course/d | Diagnosis | ΔFMA-UE | Side | Description |
| --- | --- | --- | --- | --- | --- | --- | --- |
| BCI1 | 1 | 58 | 95 | 2 | 4 | L | BG |
| BCI2 | 1 | 73 | 67 | 1 | 13 | R | PV |
| BCI3 | 1 | 61 | 279 | 1 | 8 | L | PV,PL |
| BCI4 | 1 | 41 | 349 | 2 | 1 | L | BG |
| BCI5 | 1 | 29 | 84 | 1 | 12 | L | CR |
| BCI6 | 1 | 50 | 260 | 1 | 6 | R | BG、FL,PL |
| BCI7 | 2 | 62 | 121 | 1 | 4 | R | FL,PL,TL |
| BCI8 | 1 | 47 | 48 | 1 | 14 | L | CR |
| BCI9 | 2 | 72 | 21 | 1 | 1 | R | BG |
| BCI10 | 1 | 75 | 293 | 2 | 13 | L | BG |
| BCI11 | 1 | 63 | 91 | 1 | 22 | R | BG |
| BCI12 | 1 | 52 | 118 | 2 | 7 | L | Tha |
| BCI13 | 1 | 44 | 174 | 1 | 8 | L | BG,PV |
| BCI14 | 1 | 53 | 17 | 1 | 24 | R | BG |
| BCI15 | 1 | 58 | 175 | 1 | 3 | l | BG,PV |
| BCI16 | 1 | 46 | 24 | 1 | 18 | L | CO,PV,BG |
| BCI17 | 2 | 71 | 21 | 1 | 33 | L | PV |
| BCI18 | 2 | 62 | 44 | 1 | 9 | L | BG,CR |
| BCI19 | 1 | 46 | 207 | 2 | 4 | R | BG |
| BCI20 | 1 | 43 | 14 | 1 | 46 | L | FL,PV,CC |
| BCI21 | 1 | 46 | 291 | 2 | 6 | R | BG |
| BCI22 | 1 | 60 | 80 | 1 | 5 | L | BG |
| BCI23 | 2 | 59 | 176 | 1 | 23 | L | BG,PV |
| BCI24 | 2 | 68 | 13 | 1 | 32 | L | BS |
| BCI25 | 1 | 52 | 37 | 1 | 10 | L | BG,PV |
| BCI26 | 1 | 62 | 71 | 1 | 10 | R | PV |
| BCI27 | 1 | 47 | 75 | 1 | 14 | L | BS |
| BCI28 | 1 | 71 | 41 | 1 | 11 | L | BG,PV,PL |
| BCI29 | 1 | 52 | 17 | 1 | 16 | R | FL,TL,OL,CR |
| BCI30 | 2 | 55 | 44 | 1 | 14 | L | BG,PV |
| Control1 | 2 | 78 | 33 | 1 | 6 | R | BG |
| Control2 | 1 | 54 | 145 | 1 | 0 | L | BS |
| Control3 | 1 | 66 | 64 | 2 | 3 | R | FL,CR |
| Control4 | 1 | 78 | 105 | 1 | 1 | R | BG,CR |
| Control5 | 1 | 74 | 57 | 1 | 7 | R | BG,FL,TL |
| Control6 | 1 | 52 | 150 | 1 | 7 | L | Pons |
| Control7 | 1 | 47 | 16 | 2 | 20 | R | BG |
| Control8 | 1 | 53 | 69 | 1 | 0 | L | FL,PL,BG |
| Control9 | 1 | 54 | 36 | 1 | 12 | R | BG |
| Control10 | 2 | 35 | 187 | 1 | 7 | L | BG |
| Control11 | 1 | 58 | 165 | 1 | 8 | R | PV |
| Control12 | 1 | 72 | 77 | 1 | 4 | R | BG |
| Control13 | 1 | 51 | 42 | 2 | 4 | R | BG |
| Control14 | 1 | 46 | 26 | 1 | 16 | R | BF,PV |
| Control15 | 2 | 70 | 40 | 1 | 8 | R | FL,PV |
| Control16 | 1 | 43 | 286 | 2 | 3 | L | BG |
| Control17 | 1 | 34 | 77 | 2 | 34 | L | PV,FL,PL |
| Control18 | 1 | 62 | 348 | 1 | 2 | L | TL,PL |
| Control19 | 1 | 59 | 28 | 1 | 8 | L | BG,PV,TL |
| Control20 | 1 | 74 | 275 | 1 | 4 | R | CG |
| Control21 | 2 | 22 | 20 | 1 | 4 | R | BG,CO |
| Control22 | 1 | 66 | 44 | 1 | 9 | R | FL、PV,CO |
| Control23 | 1 | 73 | 56 | 1 | 4 | L | BG |
| Control24 | 1 | 75 | 41 | 1 | 4 | R | PV |
| Control25 | 1 | 54 | 29 | 1 | 7 | R | BG |
| Control26 | 1 | 77 | 83 | 1 | 5 | L | BS |
| Control27 | 2 | 61 | 44 | 1 | 6 | L | BS |
| Control28 | 1 | 72 | 37 | 1 | 4 | L | FL,PL,TL |
| Control29 | 1 | 42 | 218 | 2 | 4 | L | BG |
| Control30 | 2 | 70 | 71 | 1 | 17 | L | BG |
| Control31 | 1 | 57 | 175 | 2 | 4 | R | BG |

diagnosis(1 Infarction,2 Hemorrhage); Abbr: BG, Basal ganglia; PV, paraventricular; CO, centrum ovale; CR, corona radiate; Tha, Thalamus; CC, corpus callosum; BS, brain stem; Pons; IC, internal capsule; FL , frontal lobe; PL, parietal lobe; TL, temporal lobe; OL, occipital lobe.
